# Evaluation of Host Cell Impurity Effects on the Performance of Sterile Filtration Processes for Therapeutic Viruses

**DOI:** 10.3390/membranes12040359

**Published:** 2022-03-24

**Authors:** Evan Wright, Karina Kawka, Maria Fe C. Medina, David R. Latulippe

**Affiliations:** 1 Department of Chemical Engineering, McMaster University, 1280 Main Street West, Hamilton, ON L8S 4L7, Canada; wrighej3@mcmaster.ca (E.W.); kawkak2@mcmaster.ca (K.K.); 2 Robert E. Fitzhenry Vector Laboratory, McMaster Immunology Research Centre, Department of Pathology and Molecular Medicine, McMaster University, 1280 Main Street West, Hamilton, ON L8S 4K1, Canada; mmedina@mcmaster.ca

**Keywords:** sterile filtration, virus purification, downstream bioprocessing, host cell protein

## Abstract

Efficient downstream processing represents a significant challenge in the rapidly developing field of therapeutic viruses. While it is known that the terminal sterile filtration step can be a major cause of product loss, there is little known about the effect of host cell impurities (DNA and protein) on filtration performance. In this study, fractions of relatively pure Vero host cell protein and DNA were spiked into a highly pure preparation of vesicular stomatitis virus (VSV). Then, the resulting solutions were sterile filtered using two commercially available 0.22 µm rated microfiltration membranes. A combination of transmembrane pressure measurements, virus recovery measurements, and post-filtration microscopy images of the microfiltration membranes was used to evaluate the sterile filtration performance. It was found that increasing the amount of host cell protein from approximately 1 µg/mL (in the un-spiked VSV preparation) to 25 µg/mL resulted in a greater extent of membrane fouling, causing the VSV recovery to decrease from 89% to 65% in experiments conducted with the highly asymmetric Express PLUS PES membrane and to go as low as 48% in experiments conducted with the symmetric Durapore PVDF membrane. Similar effects were not seen when bovine serum albumin, a common model protein used in filtration studies, was spiked into the VSV preparation, which indicates that the sterile filtration performance is critically dependent on the complex composition of the mixture of host cell proteins rather than the presence of any protein. The results presented in this work provide important insights into the role of host cell impurities on the performance of sterile filtration processes for therapeutic viruses.

## 1. Introduction

Therapeutic viruses are an important and rapidly developing class of biotherapeutics, with applications ranging from cancer treatment [1,2,3] to novel vaccines [4]. As of 2017, 38% of the new therapeutics approved by the FDA were biologics based [5], and this number is expected to continue to grow in the future. Thus, researchers have been increasingly focusing on developing efficient and scalable methods to manufacture therapeutic virus. Many advances have been made in upstream processing in recent years, such as the development of novel cell lines [6] or new bioreactor designs [7]; as a result, the bottleneck in the production process has shifted to downstream processing steps [8,9], which can represent up to 70% of the overall manufacturing costs [9]. Fortunately, progress in various areas of downstream purification, including harvest and clarification, chromatography [10], and ultrafiltration [11], has helped to relieve this bottleneck.

The terminal sterile filtration step, which is required by regulatory agencies to ensure that the final product is free of any bacterial bioburden [12], is an often-overlooked component of the downstream purification train. While sterile filtration is not an issue in many cases, some studies have reported high losses for lentivirus [8], influenza virus [13], enterovirus [14], and rhabdovirus [15] during this process. In situations where significant virus loss occurs during sterile filtration, alternative strategies can be applied, such as the aseptic processing of a virus [16] or reorganizing the downstream processing train [17]. However, this is not desirable due to cost and complexity. For some larger viruses, such as herpes virus (~200 nm) [18] and vaccinia virus (~250 nm) [19], sterile filtration is particularly challenging due to their size being similar to the rated pore size of sterile filtration membranes (0.22 µm). In all these cases, it is critical to understand the factors that influence the sterile filtration of viruses, as doing so is key to minimizing losses.

Clarification is a common membrane microfiltration unit operation that has been thoroughly studied [20]. However, key considerations, such as relative virus and impurity content, the degree of fouling experienced during filtration, and solution components, render it significantly different from sterile filtration. Similarly, many studies have investigated the removal or retention of viruses using ultrafiltration membranes [21,22,23] but few have examined the filtration of viruses with respect to maximizing transmission and throughput. Some factors that are known to influence the sterile filtration of viruses include the presence of aggregates [24,25], the membrane material and structure [15], and the solution conditions [26]. In general, two mechanisms govern the retention of viruses by the membrane: size exclusion and adsorption. As the ratio between the virus particle size and the membrane pore size decreases, it becomes more difficult for the virus to be transmitted though the membrane and more likely that it will be retained [27,28]. For effective sterile filtration, a 0.22 µm pore size rating is required based on challenge tests with Brevundimonas diminuta [29]. However, the selection of different membrane structures (symmetric vs. asymmetric, pore geometry, etc.) can still influence particle retention and membrane fouling [30,31]. In addition, viruses may adsorb directly to the membrane though a combination of electrostatic and hydrophobic effects; this is influenced by membrane chemistry, the pH and ionic strength of the solution, and the presence of any additives or other components in the solution [26,32,33]. For biopharmaceutical applications, virus formulation buffers are often precisely optimized in order to maximize virus stability [34,35], which leaves little room for modifications aimed at improving filtration performance.

One modification that can be made to improve the filtration process is to minimize the amount of residual protein and DNA impurities. DNA impurities can originate from host cells in the culture system or, if applicable, helper components such as other viruses or plasmids [36]; similarly, protein impurities are derived from host cells or are present in culture media components (i.e., fetal bovine serum) [36]. Guidelines relating to these impurities are typically strict and evaluated on a case-by-case basis [37], depending on the risks they pose. Particular concerns relating to product safety include the oncogenicity of residual DNA and the immunogenicity of residual proteins [36,38]. Although impurities are typically removed during downstream processing prior to sterile filtration, some small residual amounts can remain. This can be problematic, as small residual amounts of DNA have been shown to mediate aggregation in adenovirus and lead to reduced recovery (ratio of infectious virus in the filtrate to infectious virus in the feed) after sterile filtration [24,25].

To the best of our knowledge, no other prior work has investigated how small amounts of residual host cell impurities affect the sterile filtration performance of therapeutic viruses. However, some insight can be gained from more mature biopharmaceutical processes, such as the production of monoclonal antibodies. Studies using monoclonal antibodies have shown that numerous species of host cell proteins are still present in final formulations, even after downstream purification [39,40]. These residual proteins are a diverse population that possess a range of molecular weights, isoelectric points, and hydrophilicities [41], as well as specific properties, such as charge, structure, and reactivity, that have all been shown to influence membrane fouling [42]. Furthermore, studies in related areas have shown that residual DNA can mediate the aggregation of proteins, thus leading to membrane fouling [43], and that protein aggregates can form nucleation sites on membranes, which contributes to further fouling [44,45].

Therefore, residual host cell proteins and DNA, either alone or in combination, may affect membrane fouling during the sterile filtration of therapeutic viruses. The present study consists of a series of small-scale sterile filtration tests using viruses prepared with a defined amount of host cell protein and DNA impurities. These conditions were achieved by first producing a highly pure virus batch via sucrose-gradient (SG) ultracentrifugation and then spiking host cell protein or DNA into the virus preparation. The overall filtration performance was then assessed by monitoring the changes in transmembrane pressure (indicative of fouling) during filtration and measuring the amount of virus recovered after filtration. Since SG ultracentrifugation cannot be scaled up for large-scale manufacturing [9,46], hydrophobic interaction membrane chromatography (HIC) was used as an alternative approach for purifying a batch of virus and comparing the performance of sterile filtration on these samples. Vesicular stomatitis virus (VSV), a type of rhabdovirus, was selected as a model therapeutic virus for this study for its potential as an oncolytic virus [47] and as it possesses a bullet-shaped geometry with a relatively large size of ~70 nm in width and ~200 nm in length [48], which may present an additional challenge for typical sterile filters with a pore size of 0.22 µm. Static adsorption studies were subsequently performed to develop a more in-depth understanding of the interactions between the virus, the impurities, and the membrane.

There is a need to improve current manufacturing protocols to maximize virus recovery during 0.22 µm filtration, and an investigation of the influence of host cell impurities on filtration performance will contribute to optimizing therapeutic virus downstream processing and enabling greater production of many next-generation biopharmaceuticals.

## 2. Materials and Methods

### 2.1. Production of VSV and Isolated Host Cell Impurities

**Figure 1:**
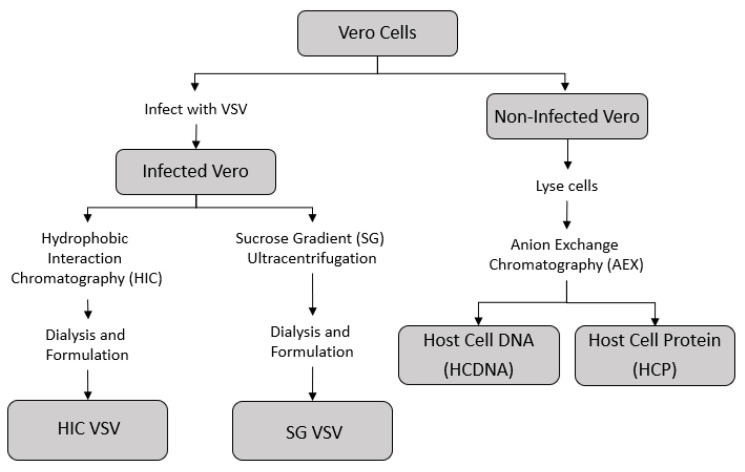
Outline of the process by which the different feed solutions for the sterile filtration experiments were prepared. The supernatant from infected Vero cells containing the vesicular stomatitis virus (VSV) was collected and purified using either hydrophobic interaction chromatography (HIC VSV) or sucrose-gradient ultracentrifugation (SC VSV)—see Table 1 for the corresponding amounts of host cell impurities in each purified VSV solution. Concentrated stocks of host cell DNA and host cell protein were prepared from a non-infected Vero cell culture using an anion exchange chromatography process.

A series of purification techniques were used to produce two sub-batches of VSV, one purified via sucrose-gradient ultracentrifugation (SG VSV) and one purified via HIC (HIC VSV). Isolated host cell protein (HCP) and host cell DNA (HCDNA) were also prepared from Vero cell lysates. Figure 1 shows a schematic of the different methodologies employed.

The Vero cells (ATCC CCL-81) used in this work were provided by the Robert E. Fitzhenry Vector Laboratory at McMaster University and were cultured using Dulbecco’s Modified Eagle Medium (DMEM) supplemented with 10% fetal bovine serum (FBS) and 1% L-glutamine (Gibco, Thermo Fisher, Waltham, MA, USA). The cells were maintained in 150 cm^2^ cell-culture flasks (Corning, NY, USA), which were incubated at 37 °C with 5% CO_2_ and passaged every 3–4 days. To expand the cells to produce uninfected Vero or VSV cultures, 15 cm diameter tissue-culture-treated dishes (Corning, NY, USA) were seeded at an approximate density of 5 × 10^5^ cells/cm^2^ and grown to confluence. The Vero cells were spit into new plates at a 1:2 ratio and were incubated for 24 h. After this time, the media was removed, the cells were washed with phosphate buffered saline (PBS), and new FBS-free DMEM was added, followed by the addition of 1 mL of VSV (Indiana strain, recombinant expressing GFP [49], provided by the Robert E. Fitzhenry Vector Laboratory) at a multiplicity of infection (MOI) of 0.1. The cells were then allowed to incubate for another 24 h, after which the cell supernatant containing virus was harvested.

The cell supernatant was portioned into 50 mL tubes, centrifuged (Beckman Coulter Allegra 6R, Brea, CA, USA) at 1400× *g* and 4 °C for 15 min to pellet any cell debris, collected, and then clarified via 0.45 µm bottle top vacuum filtration (Nalgene Rapid Flow, Thermo Fisher, Waltham, MA, USA). To prevent virus aggregation, a 0.5 M EDTA (Gibco, Thermo Fisher, Waltham, MA, USA) solution (pH 8.0) was added at a ratio of 1:25. To prepare the SG VSV samples, the filtrate containing the virus was then centrifuged (Beckman Coulter Avanti J-25i, Brea, CA, USA) using a JLA-10.500 rotor (Beckman Coulter, Brea, CA, USA) at 12,200× *g* and 4 °C for 90 min to pellet the virus. The resultant supernatant was then discarded, and the virus pellet was resuspended in 1 mL of PBS. A sucrose (BioShop, Burlington, ON, Canada) gradient was created by layering 0.5 mL of 75% sucrose, 4 mL of 40% sucrose, and 4 mL of 3% sucrose in an ultracentrifuge tube (Beckman Coulter Ultra-Clear 13.2 mL, Brea, CA, USA), from which a linear gradient was created using a Gradient Master 108 (BioComp Instruments, Fredericton, NB, Canada). Next, 1 mL of resuspended virus was placed on top of the linear sucrose gradient and ultracentrifuged using a SW41 rotor (Beckman Coulter, Brea, CA, USA) at 70,800× *g* and 4 °C for 30 min. The purified virus was visible as a single band (approximately one-third of the way down the gradient) and was collected by puncturing the side of the tube with a syringe needle. The collected virus was then dialyzed (Slide-a-Lyser G2,10 kDa cut-off; ThermoFisher, Waltham, MA, USA) against formulation buffer (150 mM NaCl, 4% sucrose, and 10 mM HEPES; pH 7.4) and further diluted to a final titer of approximately 2.4 × 10^8^ PFU/mL. The final virus solution was then portioned into 7 mL aliquots and stored at −80 °C until use.

A second sub-batch of VSV supernatant was purified using HIC, a method that has been shown to be highly effective in the purification of virus particles [50,51]. First, a Sartobind Phenyl nanocapsule (Sartorius, Göttingen, Germany) containing 3 mL of membrane was attached to an NGC medium-pressure liquid chromatography system (BioRad, Hercules, CA, USA). Next, virus-containing supernatant was prepared and clarified according to the above-described procedure and then mixed with buffer containing 10 mM HEPES and 3.6 M ammonium sulfate (Millipore Sigma, Burlington, MA, USA). The final solution consisted of 19% buffer and 81% virus supernatant, with an ammonium sulfate concentration of 700 mM and pH of 7.4. The nanocapsule was equilibrated with 10 membrane volumes of equilibration buffer (10 mM HEPES, 4% sucrose, and 700 mM ammonium sulfate; pH 7.4) at a flow rate of 9 mL/min, which was maintained throughout the experiment. Following equilibration, 10 mL of the prepared sample was applied to the capsule using the sample pump, followed by a wash step with 10 membrane volumes (30 mL) of equilibration buffer to remove any unbound impurities. Finally, the virus was eluted from the membrane via a single step change to an ammonium-sulfate-free buffer solution (10 mM HEPES and 4% sucrose; pH 7.4). During the run, conductivity and UV absorbance at both 260 nm and 280 nm were continuously monitored. The 6 mL fraction containing the largest portion of the virus was collected, dialyzed against formulation buffer (as described above), and stored in aliquots of approximately 7 mL at −80 °C.

Isolated host cell impurities were produced using a non-infected Vero cell culture. Confluent cells were detached from a 150 cm^2^ cell-culture flask, resuspended in 10 mL of low-ionic-strength buffer, i.e., Buffer A (10 mM HEPES and 4% sucrose; pH 7.4), and centrifuged at 1400× *g* for 15 min in order to pellet the cells. The supernatant was then discarded, and the cell pellet was subjected to a 3× freeze–thaw process consisting of a cold bath in 95% ethanol and dry ice and a warm bath at 37 °C. Following the freeze–thaw process, the lysed cell pellet was diluted in another 10 mL of Buffer A and centrifuged for 15 min to pellet any cell debris, and the supernatant was collected. Host cell protein and DNA were purified from this mixture using an adapted version of a previously documented method [51] and a laterally fed membrane chromatography (LFMC) device containing 1 mL of Sartobind Q membrane (Sartorius, Göttingen, Germany), which is a strong anion-exchange membrane. Briefly, the membrane was first equilibrated with Buffer A and the cell lysate was then loaded onto it using a 5 mL loop. A wash step with Buffer A was applied after loading the sample to wash any unbound material. Next, a stepwise elution profile was applied by mixing proportions of Buffer B (10 mM HEPES, 4% sucrose, and 2 M NaCl; pH 7.4) and Buffer A to generate steps of 450 mM, 600 mM, and 800 mM NaCl. The purpose of these steps was to elute protein, a mixture of protein and DNA, and DNA, respectively. From previous experiments, a 2-step elution proved insufficient for effectively separating pure host cell protein and DNA, and therefore the 3-step elution process was necessary to remove any protein and DNA co-eluting at similar intermediate ionic strengths. In each step, 6 membrane volumes (6 mL) were eluted and collected in 3 fractions of 2 mL. A constant flow rate of 5 mL/min was used throughout the process. During the run, conductivity and UV absorbance at both 260 nm and 280 nm were continuously monitored.

### 2.2. Sterile Filtration

Sterile filtration experiments were performed on a small-scale constant flux system, with flow being driven using a syringe pump (Harvard Apparatus PhD Ultra, Holliston, MA, USA) with a 10 mL syringe (Becton-Dickinson, Franklin Lakes, NJ, USA). During operation, a digital pressure transducer (Omega PX409, Biel, Switzerland) was used to measure the transmembrane pressure (TMP) relative to the atmospheric pressure. The resultant data were normalized against the starting TMP value (i.e., TMP_0_) to account for variance in membrane permeability and was averaged over a 0.25 mL interval to reduce the small degree of oscillations introduced by the syringe pump. Silicone tubing (Cole-Parmer Masterflex L/S 14, Vernon Hill, IL, USA) was used to connect the syringe to a cross junction, which was also connected to the pressure transducer and a polycarbonate membrane housing (Cole Parmer, Vernon Hill, IL, USA) with 0.5 cm^2^ of effective filtration. All tubing connections were secured using polypropylene Luer-lock fittings (McMaster-Carr, Elmhurst. Il, USA). This study used either hydrophilic polyvinylidene fluoride (PVDF, Durapore, Millipore Sigma, Burlington, MA, USA) membranes or hydrophilic polyether sulfone (PES, Millipore Express PLUS, Millipore Sigma,, Burlington, MA, USA) membranes, both of which are designed for the sterile filtration of protein or other biological solutions and have a rated pore size of 0.22 µm. It is worth noting that the PES membrane has an asymmetric pore structure, with pores being more open on the upstream side of the membrane, while the PVDF membrane is symmetric and has a consistent pore size throughout the depth of the membrane; SEM images of the membrane top, bottom, and cross section are shown in Figure S1. All fittings, tubing, and membranes were sterilized in an autoclave at 121 °C for 30 min before use.

The assembled system was first pre-wet using a syringe loaded with formulation buffer and then the membrane permeability was determined by measuring the transmembrane pressure at various volumetric flow rates, ranging from 0.1 to 10 mL/min. A 7 mL aliquot of VSV was thawed and, if necessary, spiked with host cell protein and/or DNA. For tests using bovine serum albumin (BSA, BioShop, Burlington, ON, Canada) as the spiking impurity, a 1 mg/mL stock solution was prepared in formulation buffer and then spiked into the VSV. The VSV solution was incubated at 37 °C for 1 h to allow any potential interactions between the virus and impurities to take place. The VSV solution was then loaded into a new syringe, which was then connected to the pump and the pre-wet tubing and membrane system. A total of 5 mL of VSV solution was then passed though the membrane at a flow rate of 0.15 mL min^−1^ (flux of 0.3 mL min^−1^ cm^−2^). Next, the filtrate and the remaining feed solution in the syringe were collected and stored at −80 °C for future analysis. Finally, the membrane was removed from the polycarbonate membrane holder, gently washed in Milli-Q water, and fixed in 1% glutaraldehyde for 30 min. All experiments were performed in duplicate using identical conditions, with all reported data representing the average of the two tests unless otherwise mentioned.

### 2.3. Static Adsorption

A 7 mL aliquot of VSV was thawed and further divided into 0.5 mL samples. Each 0.5 mL sample was placed into a separate 1.5 mL Eppendorf tube and then spiked with impurities as described above. An individual membrane (1.33 cm^2^ surface area) was cut into small pieces using a razor, and the different VSV solutions were then incubated for 1 h at room temperature on an orbital shaker at approximately 60 RPM. Concurrently, spiked VSV solutions with no added membrane pieces were prepared and incubated as a control. The resultant supernatant of each solution was collected and stored at −80 °C for future analysis.

### 2.4. Assays

DNA concentration was measured using a Quant-iT PicoGreen dsDNA Kit (Invitrogen) in accordance with the manufacturer’s instructions. In brief, a 10 µL aliquot of each sample was diluted in 40 µL of TE buffer, which was then mixed with 50 µL of a working reagent in a half-area black 96-well microplate (Perkin Elmer, Waltham, MA, USA), followed by incubation for 5 min in a light-free environment. The fluorescence of the samples was measured at 520 nm emission and 480 nm excitation using a SpectraMax i3 (Molecular Devices, San Jose, CA, USA) plate reader, and the DNA concentration was calculated in relation to a 1–1000 ng/mL Lambda DNA (Roche, Basel, Switzerland) calibration curve.

Protein concentration was measured using a Micro BCA Protein Assay Kit (Thermo Fisher, Waltham, MA, USA) in accordance with the manufacturer’s instructions. In brief, a 100 µL aliquot of each sample was mixed with 100 µL of a working reagent in a clear 96-well microplate (Corning, NY, USA) and incubated at 37 °C for 2 h. The absorbance of the samples was measured at 562 nm using a plate reader, and the protein concentration was calculated relative to a 1–40 µg/mL BSA (Thermo Fisher, Waltham, MA, USA) calibration curve. If necessary, the samples were serially diluted prior to measurement.

VSV titer was measured using a plaque assay. Vero cells were cultured using the above-described method and seeded into a 6-well plate (Corning, NY, USA) at approximately 6 × 10^5^ cells per well and incubated for 24 h. Virus samples were serially diluted 10-fold into supplemented DMEM, which was followed by the addition of 100 µL to each well (2 dilution levels, each in triplicate). The infected wells were incubated for 1 h at 37 °C and 5% CO_2_, with manual rocking every 15 min. An agarose overlay solution consisting of 0.5% agarose and 10% FBS in DMEM was prepared and kept warm at 40 °C; 2 mL of this overlay solution was added to each well and allowed to solidify before the wells were incubated for another 24 h. After this incubation period, the cells were fixed for 1 h at room temperature by adding 1 mL of 3.7% formaldehyde to each well. The agarose plugs were then manually removed from the wells, and the fixed cells were stained by adding 1 mL of 0.1% crystal violet in 20% ethanol to each well and leaving it for 10 min. Excess crystal violet stain was aspirated, and the wells were then washed under a gentle stream of water. Finally, the visible plaques (transparent spots on the cell layer) were counted in order to calculate the number of plaque-forming units per mL (PFU/mL) accounting for the dilution factor.

DNA fragment length was determined using an automated electrophoresis platform (Agilent TapeStation, Genomic DNA ScreenTape, Agilent Genomic DNA Reagents, Santa Clara, CA, USA). Specifically, the DNA strand length analysis consisted of a comparison of the DNA from the unpurified Vero cell lysate and the AEX chromatography-purified DNA that was used in the spiking experiments. The DNA was prepared for analysis by first concentrating it approximately 30-fold and then buffer exchanging it into 10 mM Tris-HCl, pH 8.5, 0.1 mM EDTA using a Genomic DNA Clean & Concentrator spin column kit (Zymo Research, Irvine, CA, USA) as per the manufacturer’s instructions.

Protein size analysis was performed using SDS PAGE gel and Coomassie Blue staining. As with the DNA strand length analysis, the protein size analysis compared protein from the unpurified Vero cell lysate and the AEX-chromatography-purified protein used in the spiking experiments. First, 10 µg of each protein sample was mixed 1:1 with Laemmli sample buffer (BioRad, Hercules, CA, USA) and heated in boiling water for 2 min. This was followed by separation on 8–16% acrylamide tris-glycine gel (Mini-PROETAN, BioRad, Hercules, CA, USA); a protein ladder (Chameleon Duo Pre-Stained Protein Ladder, LI-COR Biosciences, Lincoln, NE, USA) was included as a reference. After separation, the gel was gently washed for 10 min in deionized water, followed by staining with Coomassie Blue (0.02% Coomassie Brilliant Blue G-250, 10% ammonium phosphate, 20% methanol, and 2% phosphoric acid). The gel was incubated for 2 h in the staining solution with gentle shaking, then rinsed with deionized water, de-stained for 10 min in 20% methanol, and finally imaged using a ChemiDoc MP (BioRad, Hercules, CA, USA) gel-imaging system.

### 2.5. Scanning Electron Microscopy

Segments were cut out of fixed membranes using a razor and mounted onto specimen stubs with carbon tape. The samples were then sputter coated (Polaron E5100, Hertfordshire, UK) with gold for 60 s under vacuum conditions under a current of 20 mA; this resulted in the application of a layer approximately 24 nm thick. The samples were then imaged using a Vega II LSU (Tescan, Brno, Czech Republic) instrument operating at an acceleration voltage of 20 kV. 

## 3. Results and Discussion

### 3.1. Production of Purified VSV Batches and Host Cell Impurities

VSV was first purified via hydrophobic interaction chromatography (HIC). The solution conditions were selected to ensure that the VSV was selectively bound to the membrane and that impurities were washed away (appearing as the first peak in the chromatogram shown in Figure S2). The VSV was then eluted through a step change to buffer without ammonium sulfate, resulting in the second peak in the chromatogram. HIC purification combined with dialysis buffer exchange resulted in a final protein concentration of 24.5 µg/mL and a final DNA concentration of 20.7 ng/mL, corresponding to a removal of 99.7% of the protein and 90.7% of the DNA from the VSV solution. The purification of VSV via sucrose-gradient (SG) ultracentrifugation and dialysis buffer exchange resulted in a final protein concentration of 1.24 µg/mL and a concentration of DNA below the 1 ng/mL detection limit of the assay used, giving a 99.95% protein and a minimum of 99.6% DNA removal. The minimal 1.24 µg/mL of protein is likely a combination of residual host cell impurities and the viral proteins themselves, given previously measured values of PFU/µg protein [52]. Relative to this highly pure SG VSV preparation, the HIC-purified VSV contained approximately 20 times more residual host cell protein with significantly more host cell DNA, and it was theorized that these increased levels of impurities would lead to measurable differences in sterile filtration performance.

**Figure 2:**
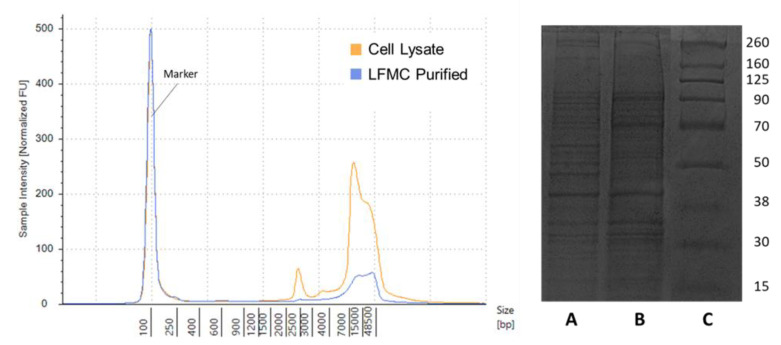
Analysis of the size distribution of purified fractions of host cell DNA and host cell protein (from AEX chromatography) obtained from Vero cells. (**Left**) Electropherogram from Agilent TapeStation analysis of strand length of host cell DNA from the crude cell lysate and from the cell lysate purified using anion exchange (AEX) chromatography (identified as Peak 4 in Figure S3). (**Right**) Image from SDS PAGE analysis of host cell protein from the crude cell lysate (Lane A); the host cell protein purified using AEX chromatography, which is identified as Peak 2 in Figure S3 (Lane B); and Chameleon Duo pre-stained protein ladder (Lane C). The original electropherogram and gel image are available in Supplementary Material as Figure S4.

Finally, AEX membrane chromatography was employed to isolate host cell protein and DNA. As previously demonstrated, this method is highly effective for separating biological components [53]. After the cell lysate containing host cell proteins and DNA was applied to the membrane under low ionic strength, the different components were selectively eluted by increasing the ionic strength in a stepwise fashion. As shown in Figure S3, a step to 450 mM eluted a solution largely consisting of pure host cell protein, with a concentration of 941 µg/mL. While there was still a small amount of residual DNA in the protein elution, the relative concentration was not high enough to not cause a significant change in the DNA concentration when creating the VSV samples spiked with protein. An intermediate step to 600 mM resulted in the elution of a mixture of protein and DNA, while a third step to 800 mM generated a highly pure host cell DNA solution (Figure S3), with a concentration of 397 ng/mL and no detectable protein. The compositions of the different eluted fractions are summarized in Table S1. To investigate how AEX chromatography purification affected the properties of the host cell protein and DNA, size analysis of the two biomolecules was conducted using electrophoretic separation. An Agilent TapeStation kit was used to generate a profile of the DNA fragment size distribution; SDS PAGE and Coomassie Blue staining were used to separate and visualize the major protein bands. The Vero cell lysate DNA consisted of a minor peak at 2400 bp and a large, broad peak from approximately 5000 bp to 50,000+ bp (Figure 2). While the minor peak was eliminated following AEX purification, the major broad peak was largely retained. The purified DNA likely consisted of a distribution of large genomic DNA fragments. Both the protein from cell lysate and the purified protein produced numerous protein bands in the 15 to 260 kDa size range. While the exact protein population shifted during purification, the purified host cell protein still contained a variety of proteins. In other downstream processing studies, impurities for spiking have been prepared via anion-exchange chromatography [54], microfiltration and diafiltration [55], flow-through from protein A chromatography [40], and simply directly spiking culture supernatant [56]. The methods described here are advantageous as the DNA and protein impurities have been individually isolated, allowing for their effects to be discriminated and studied individually.

The isolated host cell protein and the host cell DNA were then spiked into the SG VSV, resulting in the different solutions summarized in Table 1. Since HIC VSV had a higher degree of impurities compared with SG VSV, the impurity levels in the HIC VSV were used as targets when spiking the SG VSV with host cell protein and/or host cell DNA. In all cases, adding the isolated impurities resulted in a volume change of less than 5% and a less than 6% change in ionic strength. Furthermore, the addition of the impurities had no significant effect on the virus titer (*p* > 0.05).

### 3.2. Effect of Host Cell Impurities on Sterile Filtration

We sought to evaluate whether AEX-chromatography-purified host cell impurities can be used as surrogates for host cell impurities co-purified with VSV batches, as this allowed us to examine which component of host cell impurities (protein or DNA) had the greatest effect on virus recovery and fouling during 0.22 µm filtration. After purifying VSV batches using the two above-described methods (HIC and SG), we found that the HIC VSV contained higher levels of impurities compared to the SG VSV (Table 1).

**Figure 3:**
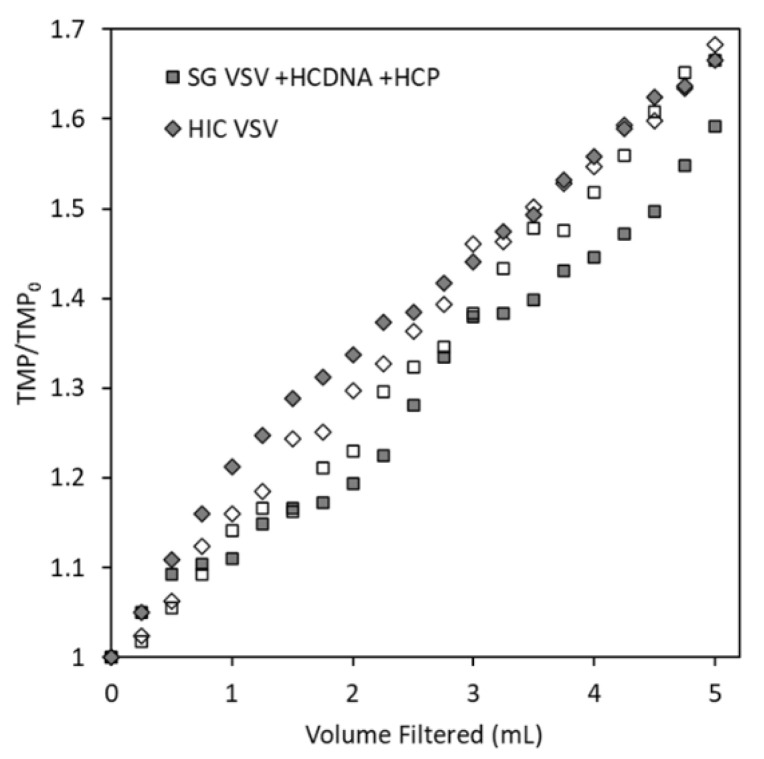
Transmembrane pressure (TMP) profiles (reported as the ratio of the measured TMP to the initial TMP) for the constant flux filtration (0.3 mL min^−1^ cm^−2^) of two vesicular stomatitis virus (VSV) solutions (identified by the symbol shape) through the Durapore PVDF 0.22 µm microfiltration membrane. The empty and filled symbols for each shape represent the duplicate filtration tests that were conducted for each solution.

By measuring increases in the TMP during filtration and comparing the results for the spiked SG VSV and HIC VSV, we can evaluate whether spiking isolated host cell protein and DNA into the SG VSV will produce a virus solution with similar filtration characteristics as HIC VSV. As shown in Figure 3, the HIC VSV and the SG VSV spiked with host cell protein and DNA (SG VSV + HCP + HCDNA) exhibited comparable increases in the TMP during filtration, along with similar virus recoveries of 49 ± 18 and 46 ± 11% respectively. Replicate filtration tests were conducted to demonstrate the precision of the process. Spiking the DNA and protein into the final solution resulted in performance that was comparable to co-purifying the impurities alongside the VSV. Furthermore, characterization of the DNA and protein impurities (Figure 2) demonstrates their significant complexity, which would be obtained using typical protein or DNA standards. Therefore, the spiking model was deemed to be appropriate for studying the effects of residual impurities. When comparing the HIC VSV to the protein- and DNA-spiked SG VSV, the exact composition of the host cell impurities must be considered. Host cell proteins are diverse [41], and different clarification and purification methods are known to select for portions of that population [39,57]. Furthermore, AEX chromatography will inherently select for a population of host cell proteins that is more negatively charged. Despite this, the spiked impurities appear to facilitate filtration performance similar to that by impurities co-eluted with VSV during HIC purification. This and all following constant flux filtration tests were performed at 0.3 mL min^−1^ cm^−2^. While this flow rate is relatively low, it is comparable to the rates used in other sterile filtration operations [58,59] and it minimizes flux-dependent fouling [30] and potential shear forces [60].

**Figure 4:**
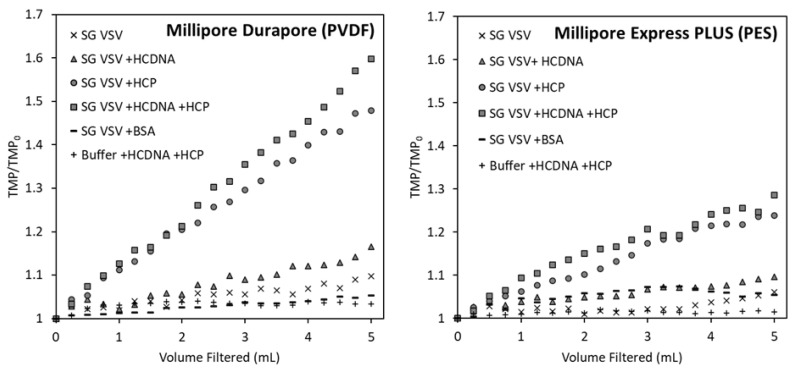
Transmembrane pressure (TMP) profiles (reported as the ratio of the measured TMP to the initial TMP) for the constant flux filtration (0.3 mL min^−1^ cm^−2^) of five vesicular stomatitis virus (VSV) solutions and one VSV-free solution through the Durapore PVDF 0.22 µm and Express PLUS PES 0.22 µm microfiltration membranes. Data points are the average of the duplicate filtration tests conducted for each solution.

Next, we wanted to investigate which impurity (protein or DNA) had a more detrimental effect during the filtration of VSV through 0.22 µm PES or PVDF membranes. As shown in Figure 4, there was a minimal increase in the TMP during the filtration of SG VSV with both the PVDF and PES membranes. Similarly, when the SG VSV was spiked with DNA (SG VSV + HCDNA), only a minor increase in the TMP was observed for both membranes. Conversely, a noticeable change was observed (a 4–5-fold increase) when the SG VSV was spiked with protein (SG VSV + HCP); however, when the SG VSV was spiked with both protein and DNA (SG VSV + HCP + HCDNA), the increase in the TMP did not significantly differ from the increase on spiking with protein alone. This indicates that while DNA concentration does not appear to play a role in membrane fouling and TMP increase during filtration, protein concentration does have an effect. For both membranes, fouling increased linearly in relation to the volume filtered, thus theoretically implicating cake formation as the main fouling mechanism [61]. In cake filtration, particulate matter collects on the surface of the membrane, building up an external layer that resists fluid flow [61]. In the filtration of biological materials, the increase in the TMP will typically follow an intermediate or standard pore blocking model [15,45,62,63] due to the adsorption and fouling of material within the membrane structure. This discrepancy could be due to the low volumes filtered and relatively mild fouling seen in these experiments, which do not allow for the observation of the full pattern of TMP increase.

No increase in the TMP was observed when the VSV was spiked with BSA at a concentration comparable to HCP. BSA is commonly used as a model protein in fundamental studies of filtration theory [45,62] and in applied bioprocessing studies [23,30], as it provides an excellent analogue for how a generic protein might behave. In this study, however, BSA was proven to be a poor representation surrogate for host cell proteins. The absence of an increase in fouling with BSA indicates that the degree of membrane fouling is linked to specific qualities of the host cell proteins, rather than the presence of protein in general. In some cases, proteins in solutions such as beef extract [32], serum proteins [28], and BSA [64] have been specifically used to prevent adsorption to membranes and fouling during virus filtration, which further suggests that fouling is likely not caused by the general presence of protein in a solution. As a control, host cell DNA and host cell protein were spiked into formulation buffer and then filtered; these tests produced little to no increase in the TMP. In other studies of protein microfiltration, the concentration and volume throughput required to achieve detectable fouling are orders of magnitude more than observed in this study [30,42]. These results demonstrate that the fouling observed when VSV is spiked with host cell protein is not simply due to the host cell proteins themselves but due to a combined interaction between the protein, the virus, and the membrane.

**Figure 5:**
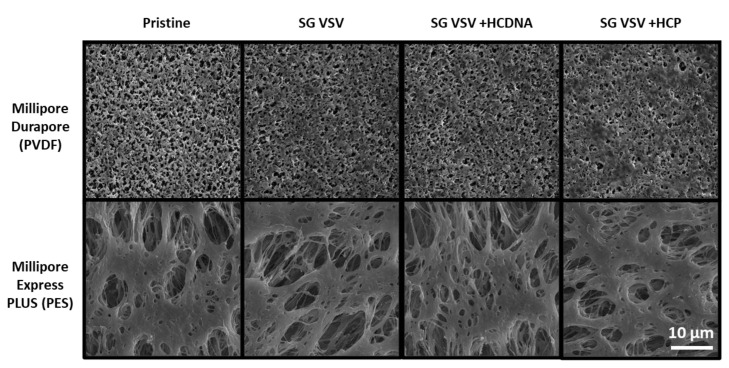
Scanning electron microscopy images (at 5000× magnification) of Durapore PVDF 0.22 µm and Express PLUS PES 0.22 µm membranes either in pristine (unused) condition or after filtering sucrose-gradient-purified vesicular stomatitis virus (SG VSV) with added host cell DNA (HCDNA) or host cell protein (HCP).

The effect of impurities can also be observed in the form of deposits on the PVDF membrane surface, as shown in the SEM images in Figure 5. As can be seen, minor fouling and build-up are present on the PVDF membrane (vs. the pristine membrane) after using it to filter SG VSV. The SG VSV spiked with host cell DNA gave a similar result. However, larger deposits can be seen following the filtrations of the SG VSV spiked with host cell protein; these deposits cover more area and clearly block some of the pores on the membrane surface. In contrast, no clear differences can be seen between the pristine PES membrane and the PES membranes that had been used to filter the different VSV solutions.

**Figure 6:**
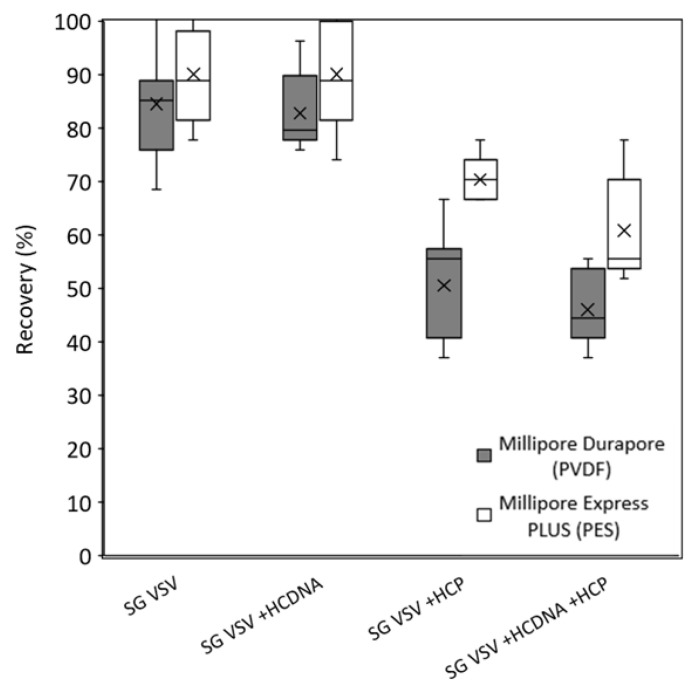
Box-and-whisker plot showing the recovery of vesicular stomatitis virus (VSV) after filtration, calculated as the ratio of filtrate to feed virus titer. Sucrose-gradient-purified VSV (SG VSV) was spiked with host cell protein (HCP), host cell DNA (HC DNA), or both host cell protein and DNA prior to filtration. Two membranes are compared, Millipore Durapore (PVDF) 0.22 µm and Millipore Express PLUS (PES) 0.22 µm. The boxes depict the interquartile range, the horizontal lines represent the median, and the cross marks represent the mean. The whiskers extending from the boxes show the maximum and minimum values measured.

The virus recovery (defined as the ratio of infectious particles in the filtrate to infectious particles in the feed) results for all the filtration tests are shown in Figure 6. When filtering the highly pure SG VSV, both membranes showed a comparable high recovery, with averages of 85 and 89% for the PVDF and PES membranes, respectively. There was no change in virus recovery between the SG VSV and the SG VSV spiked with host cell DNA for both tested membranes. However, a significant shift was seen upon spiking with host cell protein. Regardless of the presence of DNA, a significant decrease in virus recovery (*p* < 0.01) was seen, with average recoveries being reduced to 48 and 65%. In these host-cell-protein-spiked samples, the PES membrane provided a significantly higher recovery than the PVDF membrane (*p* < 0.05).

These recovery results mimic what was observed in the measurements of TMP increase during filtration (Figure 4), with both membranes having an initial low TMP increase and high recovery for the SG VSV, which upon spiking with host cell protein resulted in a higher TMP increase and lower recovery. This difference may be due to the asymmetric structure of the PES membrane, which is more open at the top surface, as this may result in more material being distributed throughout the depth of the membrane structure instead of solely on the surface. Asymmetric membranes are also known to be more resistant to fouling and give higher throughput [31], which further explains the results in Figure 4, Figure 5 and Figure 6, where the PES membrane showed improved performance by the metrics of less TMP increase, no notable visual fouling, and improved recovery.

These conclusions on the performance of the two membranes are not necessarily consistent with the existing literature. Previous work comparing the sterile filtration of a human cytomegalovirus vaccine through a selection of 0.22 µm rated membranes found that the PES Express membrane had higher fouling and lower recovery than the PVDF Durapore membrane (the exact same membranes used in this study), contrary to what was found in this study [65]. Conversely, when comparing the Millipore PVDF membrane to a PES membrane in the sterile filtration of an oncolytic Rhabdovirus, it was found that the PES membrane offered significantly better performance [15]. While not as close a comparison, in a study on the filtration of bacteriophages from wastewater though PVDF and PES membranes, no significant differences were found between the membrane types [32]. There appears to be no overarching conclusion that can be reached, and the sterile filtration performance is highly variable given the many unique properties of different virus preparations.

Recovery of protein was also measured for each filtration test, and no significant change in protein concentration was observed between the feed and the filtrate (Table S2). Even though the mass of the protein captured by the membranes was not significant, it is clear from these results that the presence of the host cell protein played an important role in determining how the VSV interacted with the membrane and the degree of fouling that was experienced.

### 3.3. Adsorption of VSV, Host Cell Protein, and DNA to Microfiltration Membranes

**Figure 7:**
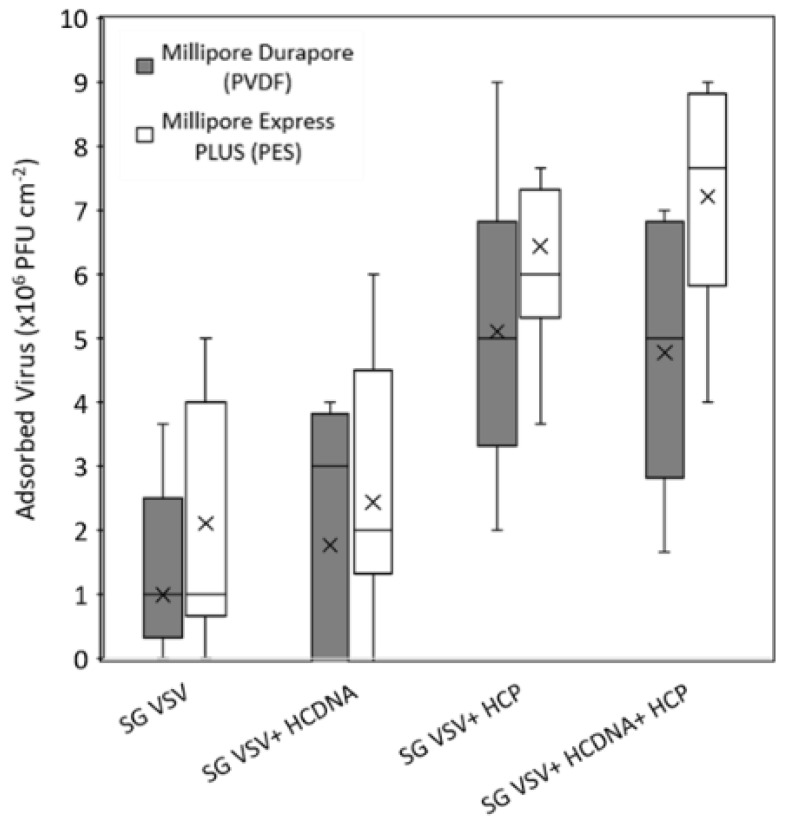
Box-and-whisker plot showing the static adsorption of sucrose-gradient-purified vesicular stomatitis virus (SG VSV) to Durapore PVDF and Express PLUS PES 0.22 µm membranes in the presence of host cell protein (HCP), host cell DNA (HC DNA), or both. Data were calculated from plaque assays and adsorption experiments performed in triplicate. The boxes depict the interquartile range, the horizontal lines represent the median, and the cross marks represent the mean. The whiskers extending from the boxes show the maximum and minimum values measured.

To better understand the interactions between VSV, host cell impurities, and the microfiltration membranes, static adsorption tests were performed. As shown in Figure 7, when no additional spiked protein was present (SG VSV and SG VSV + HCDNA tests), little to no virus adsorbed to either membrane. However, a significant amount (*p* < 0.01) of VSV adsorbed to the membrane in both conditions where the SG VSV was spiked with host cell protein (SG VSV + HCP and SG VSV + HCDNA + HCP). This again shows the important role played by the host cell protein in determining how VSV will interact with the membranes, namely VSV has a greater tendency to adsorb to the membrane when more host cell proteins are present in the solution. Loss during filtration can also be attributed to adsorption rather than pore blockage by the host cell proteins. While the PVDF and PES membranes are both considered hydrophilic and low protein binding by their manufacturers, some level of protein adsorption is still observed, even under ideal conditions [66]; however, this can vary between manufacturers and membrane units. Adsorption can be mediated though either electrostatic or hydrophobic effects [26], while host cell proteins can have a wide range of charges and hydrophilicities [41]. Furthermore, the envelope of VSV is also known to have patches of variable charge and hydrophobicity [67]. Given this, it is certainly feasible that some sort of interaction is taking place, although a mechanistic explanation would require further investigation. Another possible explanation is related to reactive groups on the host cell proteins. Studies examining membrane fouling via BSA have shown the critical role played by free thiol groups in this process [42]; notably, there are a wide range of functional proteins within the host cell protein population that may possess similar reactive groups that could mediate fouling. Due to the importance of host cell protein adsorption in the fouling process, a promising area of future work may be to use novel membrane materials and surface modifications to reduce protein interaction with the membrane surface [68]. For example, recent work has shown that the modification of PVDF microfiltration membranes with zwitterionic and PEG polymers can result in ultralow protein adsorption [69], which may be a promising approach to improving sterile filtration performance.

## 4. Conclusions

Using measurements of TMP increase, virus recovery, and microscopy, we have shown how the presence of small amounts of host cell proteins increases membrane fouling during the sterile filtration of a virus solution. At the highest levels observed, spiking with approximately 25 µg/mL host cell protein resulted in a 4.8 times greater increase in TMP and a 34% reduction in virus recovery. This effect is due, at least in part, to increased adsorption of the virus to the membrane in the presence of host cell proteins. Using static adsorption experiments, it was shown that up to 5.1 times more virus adsorbed to the membrane when host cell protein was spiked into a solution. Under the tested conditions, host cell DNA was not found to have a significant effect. It is theorized that the population of host cell proteins contains unique key proteins that mediate adsorption due to specific properties, such as charge, hydrophobicity, and reactivity. However, further work is required to validate this claim. Furthermore, this paper documented the development of and validated a method of testing membrane fouling effects based on the isolation and subsequent spiking of host cell impurities. This method could be easily used by other researchers interested in the topic. Finally, this work can benefit manufacturers of therapeutic viruses who are experiencing issues with losses during sterile filtration, as it demonstrates that improving host cell protein removal in earlier downstream purification steps can enhance sterile filtration performance.

## Figures and Tables

**Table 1:** Measurements of virus titer, protein, and DNA concentrations for the various vesicular stomatitis virus (VSV) solutions. Results are reported as the average ± the standard deviation, with BDL indicating a concentration below the assay detection limits and N/A indicating that no spiking was performed.

Starting Solution	VSV Titer (PFU/mL)	Spike	Protein (µg/mL)	DNA (ng/mL)
HIC VSV	2.2 ± 0.2 × 10^8^	N/A	24.5 ± 5.2	20.7 ± 0.75
SG VSV	2.4 ± 0.4 × 10^8^	N/A	1.24 ± 0.88	BDL
		+HCDNA	1.26 ± 0.58	24.6 ± 1.4
		+HCP	24.8 ± 4.2	BDL
		+HCDNA +HCP	23.2 ± 3.5	23.2 ± 1.1

## Data Availability

The data presented in this study are available in the article, or supplementary file.

## Supplementary Materials

The following supporting information can be downloaded at https://www.mdpi.com/article/10.3390/membranes12040359/s1: Figure S1: SEM images of the Durapore PVDF 0.22 µm and Express PLUS PES 0.22 µm membranes showing the differences in structure between the top, bottom and cross section of the membranes. Figure S2: UV absorbance (at 280 nm) and conductivity profiles for the purification of VSV using hydrophobic interaction membrane chromatography (Sartobind Phenyl). Figure S3: UV absorbance (at 280 nm) and conductivity profiles for the purification of host cell impurities using laterally-fed membrane chromatography with an anion exchange (Sartobind Q) membrane. Figure S4: Original electropherogram and unedited gel image used in Figure 2. Table S1: Protein and DNA content of the four elution peaks obtained for the purification of host cell impurities using laterally-fed membrane chromatography with an anion exchange (Sartobind Q) membrane. Table S2: Protein content of the feed and filtrate for VSV filtration experiments.
