# Supplementary material for: Evaluation of Host Cell Impurity Effects on the Performance of Sterile Filtration Processes for Therapeutic Viruses
Source: Membranes (Basel). 2022 Mar 24;12(4):359. doi: 10.3390/membranes12040359 (PMC9030567; doi:10.3390/membranes12040359)
Supplement: Supplementary file 1 [file membranes-12-00359-s001.zip › membranes-1633290-supplementary.pdf]

membranes

MDPI

Supplementary Materials

Evaluation of Host Cell Impurity Effects on the Performance of Sterile Filtration Processes for Therapeutic Viruses

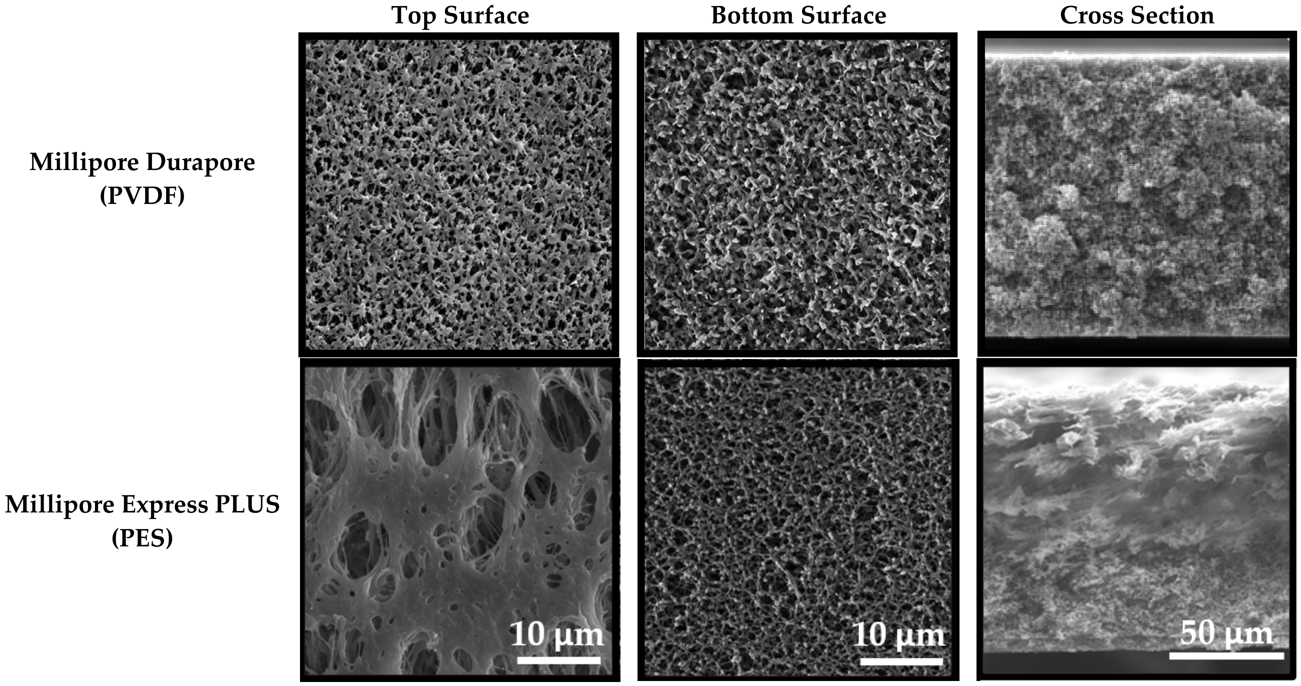

Figure S1. SEM images of the top surface (5000x magnification), bottom surface (5000x magnification), and cross section (1000x magnification) of the Durapore PVDF 0.22 µm and Express PLUS PES 0.22 µm membranes.

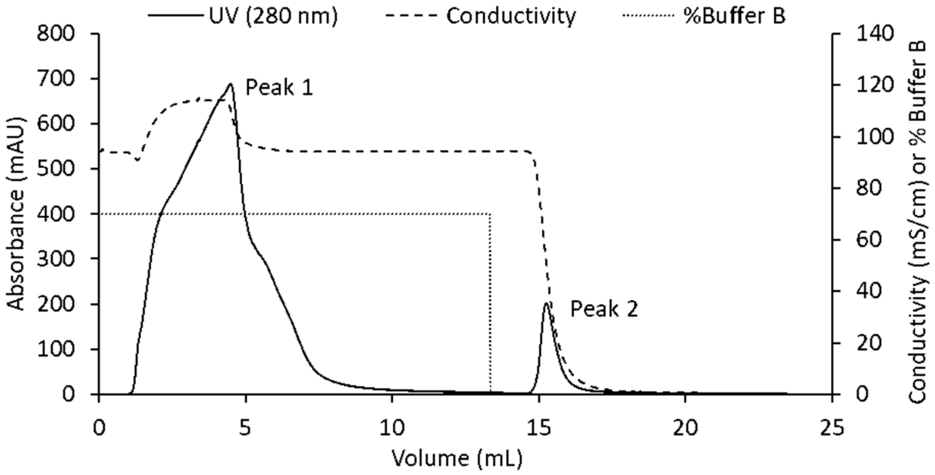

Figure S2. UV absorbance (at 280 nm) and conductivity profiles for the purification of VSV using hydrophobic interaction membrane chromatography (Sartobind Phenyl). Peak 1 corresponds to the fraction of the feed that did not bind to the membrane at the high-conductivity solution conditions (i.e. 100% Buffer B) associated with the loading step; Peak 2 corresponds to the fraction of the feed that eluted from the membrane at the low-conductivity solution conditions (i.e. 0% Buffer B). The

6 mL fraction of the eluted peak (i.e. hydrophobic interaction chromatography purified VSV (HIC VSV)) had a titer of 2.20 ± 0.23 × 10^8 PFU/mL.

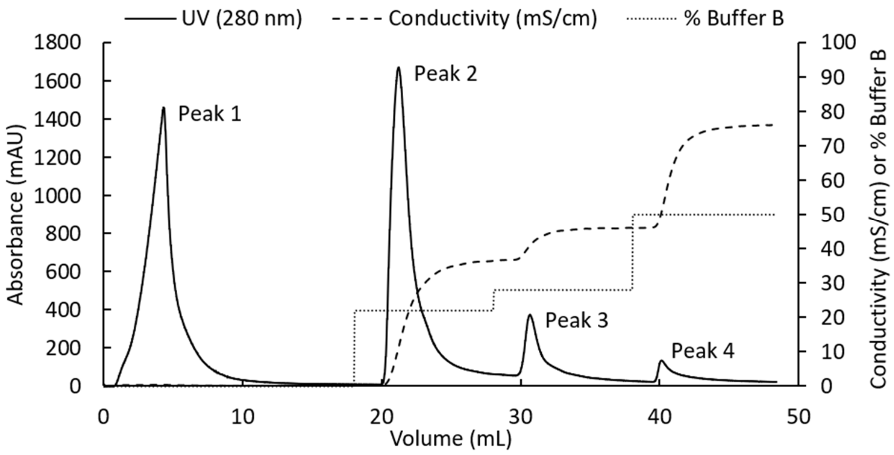

Figure S3. UV absorbance (at 280 nm) and conductivity profiles for the isolation of host cell impurities from cell lysate using anion exchange membrane (Sartobind Q) chromatography. Peak 1 corresponds to the fraction of the feed that did not bind to the membrane at the low-conductivity solution conditions (i.e. 0% Buffer B) associated with the loading step; Peak 2 corresponds to the fraction of the feed that eluted from the membrane at the solution conditions corresponding to 25% Buffer B (i.e. 75% Buffer A); Peak 3 corresponds to the fraction of the feed that eluted from the membrane at the solution conditions corresponding to 30% Buffer B (i.e. 70% Buffer A); Peak 4 corresponds to the fraction of the feed that eluted from the membrane at the solution conditions corresponding to 50% Buffer B (i.e. 50% Buffer A). As shown in Table A.1., the fractions corresponding to Peaks 2 and 4 were used to spike in controlled amounts of host cell protein and host cell DNA respectively.

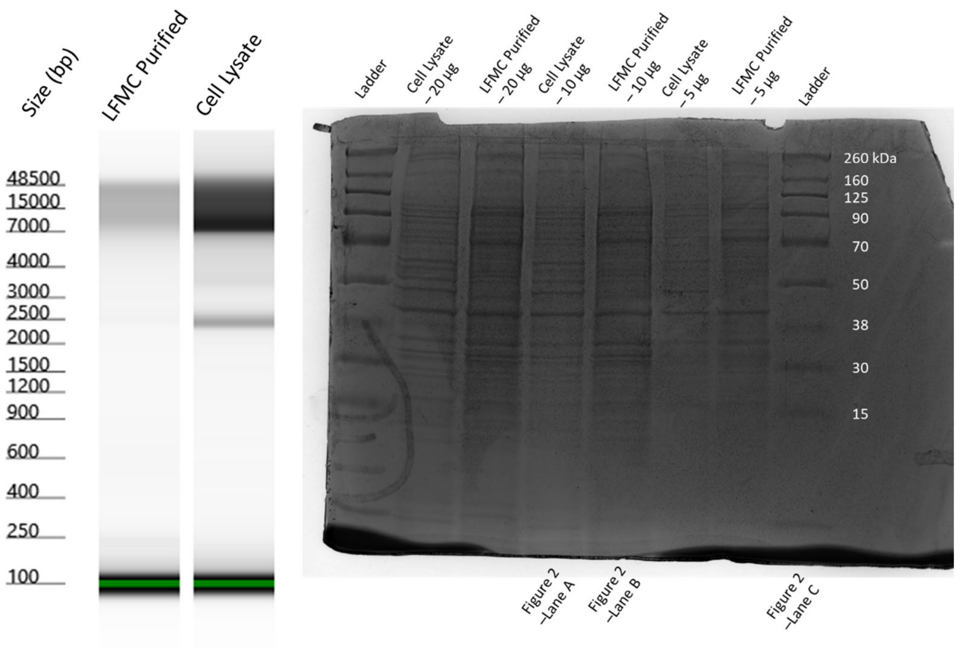

Figure S4. Original electropherogram **(Left)** and unedited gel image **(Right)** used in Figure 2. The electropherogram data was used with the Agilent TapeStation Analysis software (V 3.2) to produce the graph of signal intensity vs base pair size. The gel photograph was edited to highlight the relevant lanes.

Table S1. Protein and DNA content (measured using a Micro BCA Protein Assay Kit and Quant-iT PicoGreen dsDNA Kit respectively) of the four elution peaks identified in Figure S3 for the isolation of host cell impurities from cell lysate. BDL indicates that the concentration was below the detection limit of the assay used.

Elution Fraction	Protein (μg/mL)	DNA (ng/mL)
Peak 1	148	417
Peak 2 (HCP elution)	941	13.2
Peak 3	96.5	74.1
Peak 4 (HCDNA elution)	BDL	397

Table S2. Protein content of the feed and filtrate for VSV filtration experiments. Results reported as average ± standard deviation.

VSV Preparation	Millipore Durapore (PVDF) 0.22 μm		Millipore Express PLUS (PES) 0.22 μm	
	Feed (μg/mL)	Filtrate (μg/mL)	Feed (μg/mL)	Filtrate (μg/mL)
SG VSV	1.50 ± 0.63	1.58 ± 0.64	0.97 ± 0.68	1.14 ± 0.47
SG VSV +HCDNA	1.12 ± 0.42	1.03 ±0.88	1.39 ± 0.32	0.94 ± 0.57
SG VSV +HCP	27.5 ± 3.1	25.5 ± 2.4	22.1 ± 1.8	23.1 ± 2.8
SG VSV +HCDNA +HCP	24.7 ± 1.1	23.9 ± 2.0	23.6 ± 2.5	23.5 ± 2.1
